# JNK suppresses pulmonary fibroblast elastogenesis during alveolar development

**DOI:** 10.1186/1465-9921-15-34

**Published:** 2014-03-25

**Authors:** Sheng Liu, Harikrishnan Parameswaran, Sarah M Young, Brian M Varisco

**Affiliations:** 1 Cincinnati Children’s Hospital Research Foundation, Cincinnati, OH, USA; 2 Department of Biomedical Engineering, Boston University, Boston, MA, USA; 3 University of Cincinnati School of Medicine, Cincinnati, OH, USA; 4 Cincinnati Children’s Hospital Medical Center, 3333 Burnet Ave., MLC 2005, Cincinnati, OH 45229, USA

**Keywords:** Lung development, Elastin, c-terminal Jun kinase, Rho kinase

## Abstract

**Background:**

The formation of discrete elastin bands at the tips of secondary alveolar septa is important for normal alveolar development, but the mechanisms regulating the lung elastogenic program are incompletely understood. JNK suppress elastin synthesis in the aorta and is important in a host of developmental processes. We sought to determine whether JNK suppresses pulmonary fibroblast elastogenesis during lung development.

**Methods:**

Alveolar size, elastin content, and mRNA of elastin-associated genes were quantitated in wild type and *JNK*-deficient mouse lungs, and expression profiles were validated in primary lung fibroblasts. Tropoelastin protein was quantitated by Western blot. Changes in lung JNK activity throughout development were quantitated, and pJNK was localized by confocal imaging and lineage tracing.

**Results:**

By morphometry, alveolar diameters were increased by 7% and lung elastin content increased 2-fold in JNK-deficient mouse lungs compared to wild type. By Western blot, tropoelastin protein was increased 5-fold in JNK-deficient lungs. Postnatal day 14 (PND14) lung JNK activity was 11-fold higher and pJNK:JNK ratio 6-fold higher compared to PN 8 week lung. Lung tropoelastin, emilin-1, fibrillin-1, fibulin-5, and lysyl oxidase mRNAs inversely correlated with lung JNK activity during alveolar development. Phosphorylated JNK localized to pulmonary lipofibroblasts. PND14 JNK-deficient mouse lungs contained 7-fold more tropoelastin, 2,000-fold more emilin-1, 800-fold more fibrillin-1, and 60-fold more fibulin-5 than PND14 wild type lungs. Primarily lung fibroblasts from wild type and JNK-deficient mice showed similar differences in elastogenic mRNAs.

**Conclusions:**

JNK suppresses fibroblast elastogenesis during the alveolar stage of lung development.

## Background

Beginning at approximately 35 weeks gestation and continuing for several years postnatally [1], large saccular air spaces in the lung are progressively divided into smaller ones by secondary alveolar septa leading to a twenty-fold increase in alveolar surface area [2]. This exponential increase in gas-exchange capacity is essential to meeting the bioenergetic needs of *ex utero* growth and development. Formation of a low-compliance elastin band at the leading edge of a growing secondary alveolar septal ridge likely plays critical mechano-developmental role in alveolar septation [3-6].

Elastin is necessary for structure and function of both vascular tissues and epithelial-containing organs. It is critical to the hysteretic properties of both arteries [7] and lung [8] and to the elasticity of other epithelial organs such as skin and intestine. In infants with impaired alveolar development [9] and in animal models of the same [10-12], elastin fiber density and organization is disrupted. Regulators of elastogenesis (formation of elastin fibers) such as transforming growth factor-β [13], fibroblast growth factor-2 [14], epidermal growth factor [15], and insulin like growth factor-1 [16] are well described. With regards to signaling in the fibroblast, pro-elastogenic cytokines generally activate SMAD-signaling pathways and anti-elastogenic activate mitogen-activated protein kinases 1%3 (erk 2%1) [17]. The spatial-temporal regulation of pro- and anti-elastogenic signals is likely of critical importance since the formation of discrete elastin bands is critical to both alveolar and vascular development, and elastin experiences virtually no turnover with a half live of 74 years [18] despite expression of pro-elastogenic cytokines throughout life [19]. In the arterial vasculature, JNK signaling has been shown to suppress elastogenesis [20], but no such role has been demonstrated in the lung. We hypothesized that activation of JNK suppresses elastogenesis during alveolar lung development contributing to the localization of elastin to alveolar septal tips. To test this hypothesis, we assessed JNK activation during lung development, localized phosphorylated JNK by confocal microscopy, quantitated alveolar development and elastin localization in JNK deficient mice, and quantitated mRNA levels of elastin-associated genes in JNK deficient mouse lungs and primary fibroblasts.

## Methods

### Animal use

C57BL/6J wild-type, B6.*Mapk8*^*tm1Flv*^/J (*JNK1*^*−/−*^), B6.*Twist2*^*tm1.1(cre)Dor*^/J(*Dermo-1-cre*), and B6.*Gt(ROSA)26Sor*^*tm4(ACTB-tdTomato,-EGFP)Luo*^/J(*tomato*) mice were obtained from Jackson Laboratories. Previously described mice with a floxed *JNK1* allele (*Mapk8*^*tm2Flv*^/J, referred to as *JNK1*^*LoxP/LoxP*^) on a B6.Mapk9^tm1Flv^Mapk10^tm1Flv^/J background (hereafter referred to as *JNK2*%*3*^*−/−*^) were utilized with permission from Roger Davis at the University of Massachusetts [21]. Since JNK3 is expressed only in the heart, testis and brain [22], single *JNK2* or *JNK3* knockout animals were not utilized. All animals were housed in a barrier facility with purified air and provided purified water and autoclaved food *ad libitum*. Animal use was approved by the Cincinnati Children’s Hospital Medical Center Animal Use and Care Committee.

To localize lung JNK activity, we utilized a mesenchymal cell lineage tracing strategy with immunofluorescent staining of pJNK. *Dermo1* (a.k.a. *twist2*) is mesenchymal transcription factor expressed as early as E15.5 [23]. The “tomato” mouse constitutively expresses a membrane-bound td-tomato except in the presence of cre in which recombination excises td-tomato and eGFP is expressed. Crossing *Dermo1-cre* mice with “tomato” mice lineage traces lung mesenchymal cells with eGFP.

#### Genotyping

Mice were genotyped using the primers listed in Table 1.

**Table 1:** Genotyping primers used

**Allele**	**Common forward primer**	**Reverse primer**
JNK1-null	CCAGGCTCTCCTCATCTTCA	Wild Type-CAGCTCATTCCTCCACTCATG
		Mutant-TCACCACATAAGGCGTCATC
JNK2-null	GTTAGACAATCCCAGAGGTTGTGTG	Wild Type-CCAGCTCATTCCTCCACTCATG
		Mutant-GGAGCCCGATAGTATCGAGTTACC
JNK3-null	CGTAATCTTGTCACAGAAATCCCATAC	Wild Type-TCCAGACTGCCTTGGGAAAA
		Mutant-CCTGCTTCTC AGAAACACCCTTC
JNK1-floxed	GGATTTATGCCCTCTGCTTGTC	GAACCACTGTTCCAATTTCCATCC

### Lung tissue processing and analysis

#### Tissue procurement and processing

Mice were sacrificed by intraperitoneal injection of ketamine, xylazine, and acepromazine (100, 6, and 2 mg/kg respectively) and severing of the left renal artery. After exsanguination, the trachea was cannulated with a 22 gauge blunt tip needle and lungs isovolumetrically inflated with 4% paraformaldehyde in PBS at a pressure of 25 cm H_2_O. Lung inflation was maintained by securing a silk ligature around the trachea, and then the chest was opened. The lungs were removed and then fixed overnight at 4°C. After fixation, the lung lobes were removed from the bronchi and dehydrated by serial passage into 70% ethanol and paraffinized. Five μm sections obtained at random angles through all five lobes.

#### Lung elastin staining and quantification

Lung sections were stained for elastin using the Hart’s Staining method (PolyScientific) and elastin quantitated by previously published methods [24]. Using a Zeiss Axio Imager A.2 three 20X images were obtained from each lobe yielding fifteen images per mouse. Using MatLab (MatWorks) these images were then separated into four components by cluster analysis after defining a color spectrum and intensity values for dense elastin (arterial walls and elastin bands at septal tips), diffuse elastin (elastin in alveolar walls and bases), non-elastin tissue, and airspace. Pixel quantitation for each component was performed for each image and average values per animal used for statistical analysis. For quantitation, the fraction of each component of total tissue (dense elastin + diffuse elastin + non-elastin tissue) was utilized.

#### Morphometry

Mean alveolar diameter was calculated utilizing a published morphometric MatLab program [8,25-28], and fractional airspace was calculated using previously described methods [29].

By defining secondary alveolar crests as invaginations into distal lung airspace by tissues associated with an elastin bundle, numbers of secondary alveolar septal crests were counted per 20X field.

#### Immunofluorescence

*Dermo1-cre-tomato* mice were sacrificed at PND5 and the lungs immunostained for pJNK using an AF647 secondary antibody. Confocal images were obtained using a Nikon AR1si inverted microscope.

### Tissue culture

#### Pulmonary fibroblast isolation

Pulmonary fibroblasts were isolated from PND14 mice by previously described methods which yield >85% fibroblast purity [30,31]. Briefly, PND14 mice were sacrificed and their lungs inflated by gently instilling dispase (BD Biosciences) via the trachea and then plugging the trachea with 1% low melting point agarose. The lungs were incubated in dispase at 25°C for 45 minutes and lung tissue teased from bronchi and large vessels using sterile forceps. Dispase was neutralized using DMEM with 10% FBS and the fibroblasts were allowed to adhere in a 100 cm^2^ tissue culture dish for 1 hour at 37°C and the plate rinsed with PBS. The fibroblasts were allowed to grow in DMEM with 10% FBS and 1% Penicillin/Streptomycin and used at passage 3.

#### In vitro Cre-recombinase experiments

*JNK2*%*3*^*−/−*^ (which also has floxed *JNK1* alleles) pulmonary fibroblasts were infected at 50% confluence with 10^6^ plaque forming units of replication-deficient adenovirus expressing GFP or Cre (Vector BioLabs). Efficacy was assessed by Taqman qPCR for JNK1 mRNA (Applied Biosystems, proprietary primers). RNA and cell culture media was collected at 48 hours.

### JNK activity, protein, RNA quantification

#### JNK activity assays

For *in vivo* experiments, lung homogenate c-Jun phosphorylation was quantitated to measure JNK activity. To do so, JNK was immunoprecipitated from lung homogenates using an isoform nonspecific JNK antibody (Santa Cruz) conjugated to agarose beads. Precipitated JNK was incubated with GST-labeled c-Jun (Michal Karin, University of California at San Diego) [32] and P-32 adenosine triphosphate, the kinase solution was separated on a polyacrylamide gel, transferred to a PVDF membrane, and radiolabeled c-Jun quantitated using a Storm 860 phosphorimager.

#### Western blot

Lungs from mice of at least two different litters were snap frozen and homogenized in RIPA buffer with protease inhibitor cocktail (Sigma) using a Qiagen TissueLyser II. Protein content was determined and the homogenates were electrophoretically separated and transferred to PVDF membrane. When not frozen, lysates were kept on ice until electrophoresis. Western blot for pJNK and JNK (both isoform non-specific, 1:200 dilution, Santa Cruz) and tropoelastin (1:2000 dilution, Elastin Products Company) was performed and chemiluminisence detected using a General Electric LAS3000.

#### PCR

Lungs from mice aged E15.5 to 8 weeks were snap frozen and then homogenized in RLT buffer using a Qiagen TissueLyser II. For primary lung fibroblasts, passage two cells were seeded at 50% density and collected at 48 hours (with cells achieving 100% density). Tropoelastin, emilin-1, fibrillin-1, lysyl oxidase, fibulin-5, surfactant protein B, CD31, and GAPDH mRNA was quantitated by Taqman PCR (Applied Biosystems proprietary primers).

#### Soluble elastin quantification

Pulmonary fibroblasts were cultured for 48-hours in DMEM, 10% FBS, 1% penicillin/streptomycin and the media analyzed for elastin content using the Fastin kit (Biocolor, UK) per manufacturer instructions.

### Statistical analysis

Statistical comparisons between groups were performed using the Student two-tail *t*-test or one-way ANOVA using the Holm-Sidak method. p-values of <0.05 were considered significant.

## Results

### JNK-deficient mice have enlargement of distal lung airspaces

**Figure 1:**
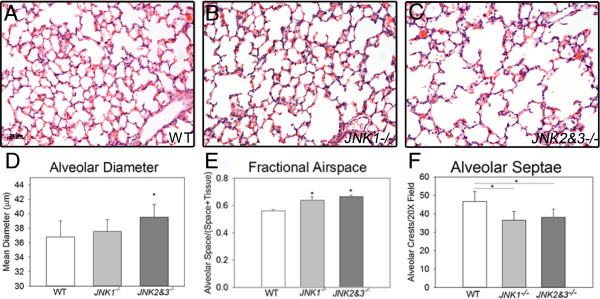
**Alveolarization is impaired in JNK-Deficient mice. (A-C)** By gross inspection of PND14 lung sections, both *JNK1*^*−/−*^ and *JNK2%3*^*−/−*^ mice had enlarged distal airspaces compared to wild type mice. Scale bar = 20 μm. **(D)** Compared to wild type lungs, *JNK2%3*^*−/−*^ lungs had larger mean alveolar diameter. **(E)** Compared to wild type lungs, both *JNK1*^*−/−*^ and *JNK2%3*^*−/−*^ lungs had increased fractional airspace. **(F)** Both *JNK1*^*−/−*^ and *JNK2%3*^*−/−*^ lungs had decreased numbers of alveolar septa per 20X field. *p < 0.05 compared to wild type by one-way ANOVA. Six animals from two different litters were used for analysis except *JNK1*^*−/−*^ which contained 3 animals from 3 different litters.

The lungs of PND14 *JNK1*^*−/−*^ and *JNK2*%*3*^*−/−*^ mice appeared to have larger airspaces with fewer alveoli as compared to wild type mice (Figure 1A-C). Mean alveolar diameter was increased by 2% in *JNK1*^*−/−*^ mouse lung and by 7% in *JNK2*%*3*^*−/−*^ lungs (Figure 1D.) Fractional airspace was increased by 14% in *JNK1*^*−/−*^ mouse lung and by 19% in *JNK2*%*3*^*−/−*^ lungs (Figure 1E). Both *JNK1*^*−/−*^ and *JNK2*%*3*^*−/−*^ mice had 20% fewer alveolar crests than wild type mice (Figure 1F). The enlarged distal airspaces and reduced numbers of secondary alveolar septal crests in JNK-deficient mice support a role for JNK in alveolar septation.

### JNK reduces lung elastin content

**Figure 2:**
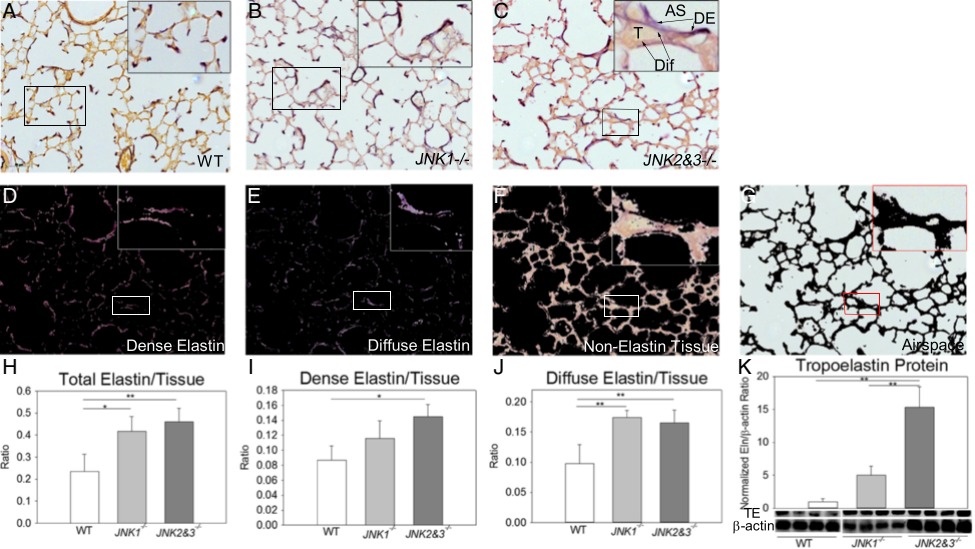
**JNK-deficient lungs have increased elastin content. (A)** PND14 wild type lungs had the typical discrete elastin bands (purple) at the tips of secondary alveolar septa with very little elastin within the tissue (yellow) of the alveolar walls and bases. Scale Bar = 50 μm. **(B-C)** Grossly, PND14 *JNK1*^*−/−*^ and *JNK2%3*^*−/−*^ lungs had increased quantities of dense elastin (DE) and more diffuse elastin (Dif) throughout the lung. T = Non-elastin tissue, AS = airspace. **(D-G)** The *JNK2%3*^*−/−*^ lung section is used to demonstrate elastin quantification. The image was separated into dense elastin, diffuse elastin, non-elastin tissue, and airspace components. The pixel counts for each separated image were used for quatitation. **(H)** Adding the dense and diffuse elastin pixel counts and normalizing to total tissue pixel count, the lungs of *JNK1*^*−/−*^ and *JNK2%3*^*−/−*^ mice had more total elastin than wild type lung. **(I)** The lungs of *JNK2%3*^*−/−*^ mice had a higher dense elastin to tissue ratio than wild type. **(J)** The lungs of *JNK2%3*^*−/−*^ mice had increased diffuse elastin content compared to wild type. For image analysis, six animals from two different litters were used for analysis except *JNK1*^*−/−*^ which contained 3 animals from 3 different litters. **(K)** Western blot for tropoelastin using PND14 lung homogenates from four mice from two different litters demonstrated increased tropoelastin in *JNK2%3*^*−/−*^ mouse lungs. *p < 0.05, **p < 0.01 by one-way ANOVA.

To test whether JNK suppresses lung elastogenesis, elastin was morphometrically quantitated in PND14 wild type, *JNK1*^*−/−*^ and *JNK2*%*3*^*−/−*^ elastin-stained lung sections. Grossly, lung elastin content was increased in *JNK1*^*−/−*^ and *JNK2*%*3*^*−/−*^ lungs compared to wild type (Figure 2A-C). As described in methods, we quantitated the number of dense elastin, diffuse elastin, tissue, and airspace pixels in elastin-stained lung sections from at least three mice from two to three different litters (Figure 2D-G). Wild type lungs contained 7% more tissue than *JNK*-deficient lungs (not significant). Normalized to total tissue, *JNK1*^*−/−*^ and *JNK2*%*3*^*−/−*^ lungs had twice the amount of total elastin as wild type lungs (Figure 2H). While *JNK1*^*−/−*^ lungs had a non-significant increase in dense elastin to total elastin ratio, *JNK2*%*3*^*−/−*^ lungs had a 1.5-fold increase compared to wild type (Figure 2I). Both *JNK1*^*−/−*^ and *JNK2*%*3*^*−/−*^ lungs had 1.7 fold more diffuse elastin than wild type lungs (Figure 2J). By Western blot of PND14 lung homogenates, *JNK1*^*−/−*^ and *JNK2*%*3*^*−/−*^ lung contained 5- and 15-fold more tropoelastin than wild type lung homogenates respectively (Figure 2K). These data support the hypothesis that JNK suppresses elastogenesis during lung development and plays a role in the localization of elastin fibers to alveolar septal tip.

### JNK activity Is increased during postnatal lung development

**Figure 3:**
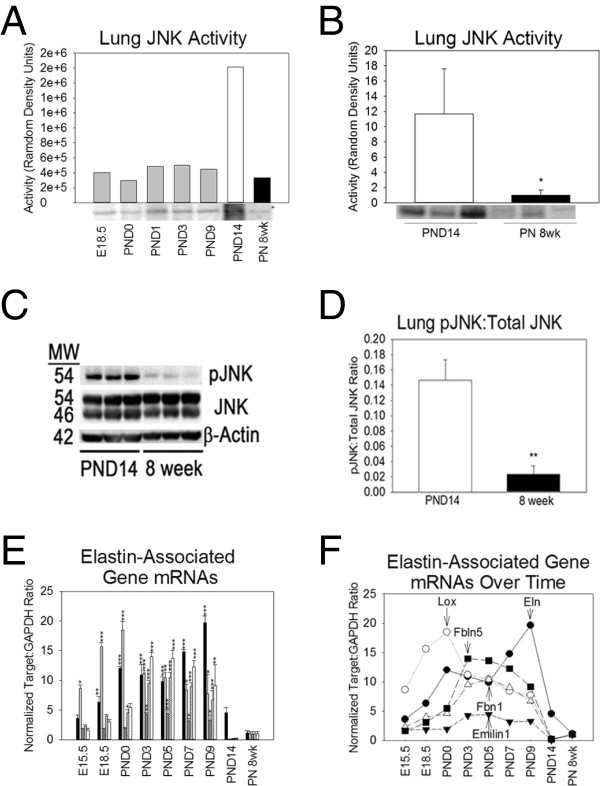
**JNK activation and elastogenesis are inversely correlated. (A)** By JNK pull down assay, JNK activity (c-Jun phosphorylation) was increased during saccular (PND1%3) and alveolar (PND9%14) stages of lung development with activity at PND14 being highest. **(B)** Comparing the lung JNK activity of mice from different litters, PND14 lungs had 11-fold higher JNK activity than PN 8 week lungs. **(C)** JNK phosphorylation was assessed by Western blot of lung homogenates from a different set of PND14 and PN 8 week mice (three mice from two different litters). PND14 mice had increased phosphorylation of the p54 JNK isoform but not the p46 isoform. No age-dependent difference in JNK isoform abundance was observed. **(D)** Western blot densitometry demonstrated a 7-fold increase in pJNK to total JNK ratio in PND14 lung homogenate compared to PN 8 week. **(E)** Tropoelastin (black bars), lysyl oxidase (gray bars), emilin-1 (dark gray bars), fibrillin-1 (dashed white bars), and fibulin-5 (white bars) mRNAs are all decreased in PND14 and PN 8 week lung compared to time points during saccular and early alveolar lung development (PND0-PND9). RNA is from lungs of three mice from at least two different litters. The noted statistical comparisons are to PN 8 weeks only but analyzed by one-way ANOVA. **(F)** A line plot of these same data demonstrates that despite different ages of peak mRNA concentration (denoted by arrows) the mRNA concentration of all five elastogenic genes decreases significantly at PND14. * p < 0.05, **p < 0.01, ***p < 0.001 by one-way ANOVA (>2 groups) or by two tail Student *t*-test (2 groups).

To determine whether JNK activity is dynamic over the course of lung development, we immunoprecipitated JNK from lung homogenates and quantitated conversion of c-Jun to phospho-c-Jun. Compared to PN 8 week lung, JNK activity was increased during the saccular and alveolar stages of lung development with the highest JNK activity in PND14 lung homogenates (Figure 3A). When confirmed in triplicate, JNK activity at PND14 was 11-fold higher than JNK activity at PN 8 weeks (Figure 3B). By Western blot, phosphorylation of the p54 isoform of JNK was increased in PND14 lung compared to PN 8 week without phosphorylation of the p46 isoform. There were no differences in total JNK protein content (Figure 3C-D). Among the molecules required for elastin fibril assembly are tropoelastin, lysyl oxidase, emilin-1, fibrillin-1, and fibulin-5. To identify an association between JNK activity and elastogenesis, mRNAs of these elastin-associated genes were quantitated at time points between E15.5 and postnatal week eight. The mRNAs of tropoelastin, lysyl oxidase, emilin-1, fibrillin-1, and fibulin-5 were all decreased during late alveolar development (PND14) compared to earlier time points (Figure 3E-F). These data demonstrate an association between decreased transcription of elastin-associated molecules and increased lung JNK activity during later alveolar developent.

### JNK activity is localized to pulmonary lipofibroblasts during alveolar development

**Figure 4:**
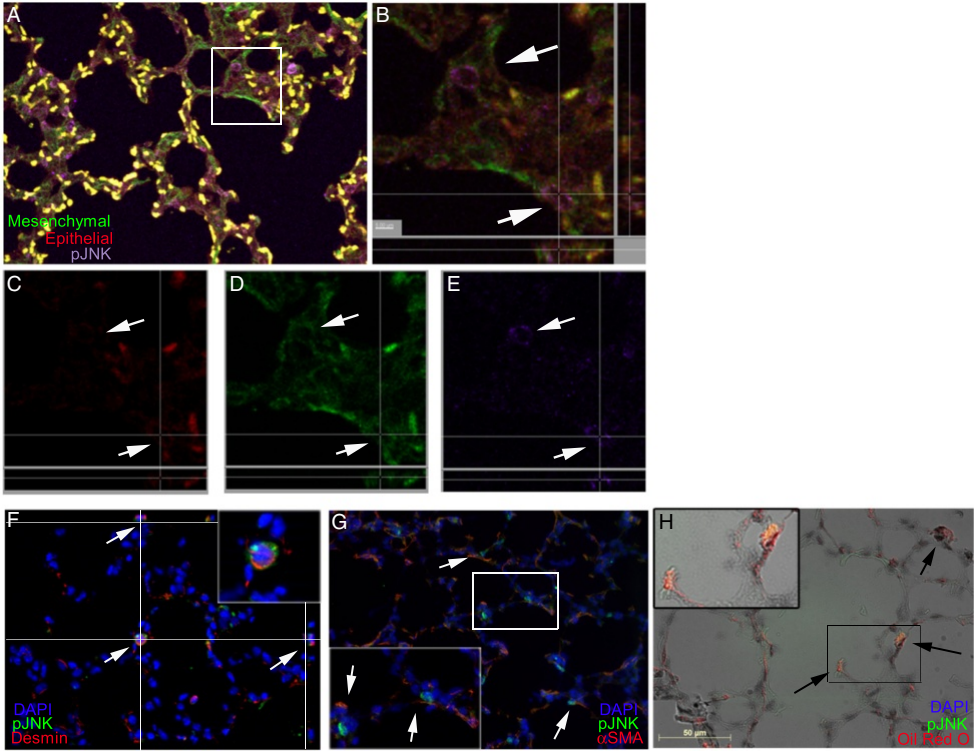
**JNK is activated in lipofibroblasts during alveolar development. (A)** By lineage tracing of lung mesenchymal cells, pJNK (purple) was located within mesenchymal cells (green), and not epithelial cells (red). 20× image. **(B)** An enlarged area of the confocal image with Z-plane sectioning showed that individual mesenchymal cells were located entirely within the green-stained mesenchymal cells. Scale bar = 5 μm. **(C-E)** Monochromatic images with z-planes demonstrated the presence of pJNK within mesenchymal cells. **(F)** Fibroblasts constitute only a subset of lung mesenchymal cells. Co-staining for pJNK and vimentin demonstrated that pJNK was localized to vimentin-positive cells although many vimentin positive cells did not contain pJNK. 40× image. **(G)** Lung myofibroblast were identified by staining for α-smooth muscle actin (αSMA). pJNK did not localize with αSMA positive cells. 40× image. **(H)** Lung lipofibroblasts were identified by Oil-Red-O staining. Using immunofluorescent imaging and differential interference contrast microscopy pJNK was located within interstitial cells having increased numbers lipid droplets but not within lipid-containing cells in airspaces (i.e. macrophages, upper right). 40× image, Scale Bar = 50 μm. Images are representative of sections from at least three different animals.

We utilized both lineage tracing and immunofluorescent co-localization techniques to determine which cell type was experiencing JNK activation. We first narrowed the range of possible cell types by staining for pJNK in PND5 mesenchymal cell lineage traced lung sections. pJNK was present in a subset of mesenchymal cells and was not activated in epithelial or hematopoietic cells (Figure 4A-E). Since pulmonary fibroblasts, endothelial cells, and pericytes are all derived from the lung mesenchyme, we performed immunostaining for desmin which is expressed in fibroblasts but not the other mesenchymal cell types. pJNK was present only desmin-positive cells (Figure 4F). Pulmonary fibroblasts can grossly be separated into the more fibrogenic myofibroblasts and less fibrogenic lipofibroblasts. Myofibroblasts express higher levels of α-smooth muscle actin (αSMA). pJNK was not located within αSMA positive cells (Figure 4G). Lipofibroblasts contain increased numbers of lipid droplets, produce less tropoelastin [31,33], and are typically located in alveolar corners [34]. In oil-red-o stained sections, pJNK localized to lipofibroblasts and not to oil-red-o positive macrophages in airways (Figure 4H). These data demonstrate that JNK activity during later alveolar development is restricted to pulmonary lipofibroblasts.

### JNK suppresses pulmonary fibroblast elastogenesis

**Figure 5:**
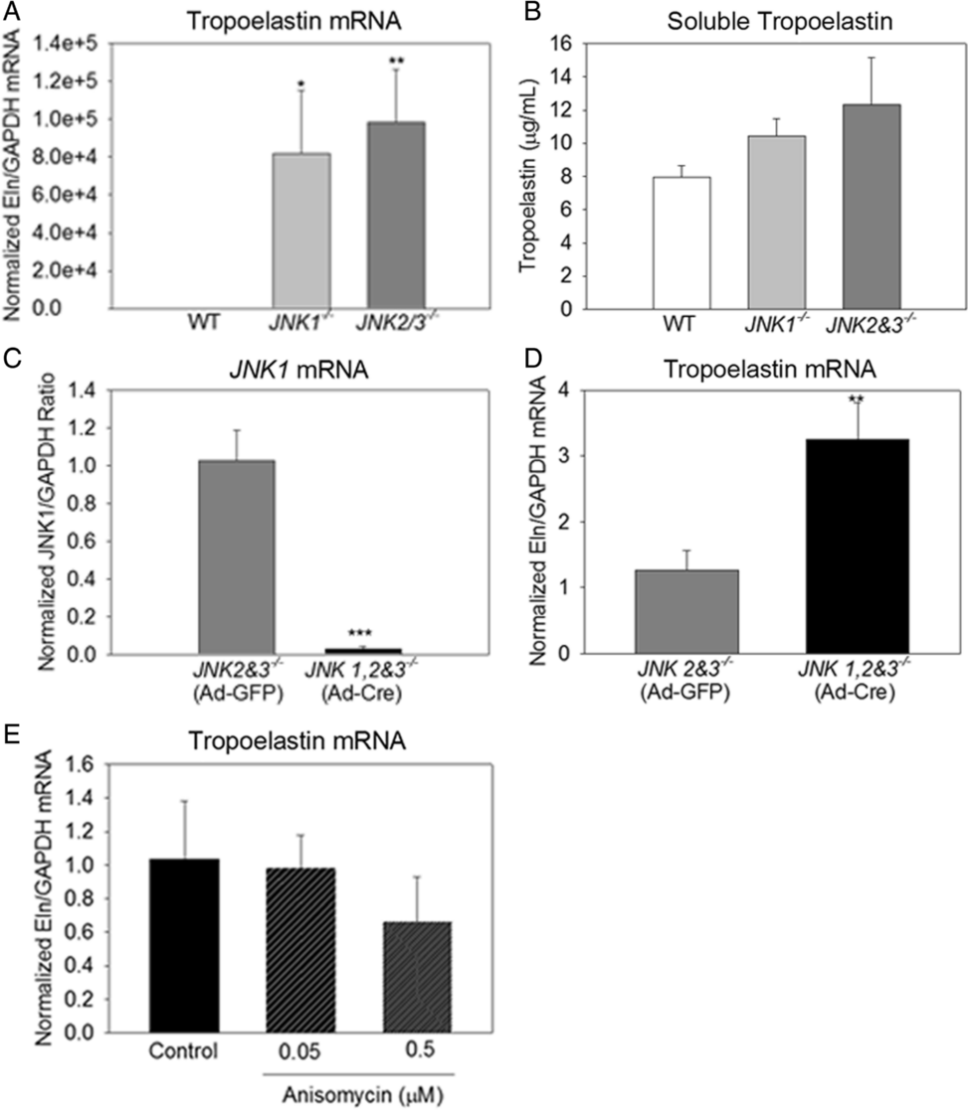
**JNK suppresses pulmonary fibroblast elastogenesis *in vitro*. (A)***JNK1*^*−/−*^ and *JNK2%3*^*−/−*^ PND14 lung fibroblasts contained more tropoelastin mRNA than wild type lung fibroblasts. **(B)** The media from JNK-deficient lung fibroblasts trended towards having more soluble tropoelastin than media from wild type lung fibroblasts. **(C)** Infection of PND14 *JNK2%3*^*−/−*^ lung fibroblasts (which also contained floxed *JNK1* alleles) with cre-expressing adenovirus (Ad-Cre) was effective at reducing JNK1 mRNA levels when compared to fibroblasts infected with a green fluorescent protein adenovirus (Ad-GFP). **(D)** Deletion of all three JNK isoforms from pulmonary fibroblasts resulted in increased tropoelastin mRNA compared to deletion of only *JNK2%3*. **E**-The JNK activator anisomycin reduced wild type PND14 lung fibroblast tropoelastin mRNA in a dose-dependent manner. *p < 0.05, **p < 0.01, ***p < 0.001 by one-way ANOVA. All analyses were performed in triplicate.

Since JNK suppresses elastogenesis in the aorta [20] and other MAP kinases regulate pulmonary fibroblast elastin synthesis [17], we tested whether JNK suppresses pulmonary fibroblast elastogenesis. Primary PND14 lung fibroblasts were cultured from wild-type, *JNK1*^*−/−*^, and *JNK2*%*3*^*−/−*^ mice. *JNK1*^*−/−*^ and *JNK2*%*3*^*−/−*^ lung fibroblasts contained 8,000-fold more tropoelastin mRNA than wild type (Figure 5A), and the media from *JNK1*^*−/−*^ and *JNK2*%*3*^*−/−*^ lung fibroblast cultures contained 50% more soluble tropoelastin (Figure 5B). All three cell types were 50% confluent at seeding and 100% confluent at collection. To test whether JNK1 and JNK2 independently suppressed pulmonary fibroblast elastogenesis, *JNK1* alleles were deleted from *JNK2*%*3*^*−/−*^ lung fibroblasts using a cre-expressing adenovirus (Ad-Cre). JNK1 mRNA was reduced 35-fold in Ad-Cre infected fibroblasts compared to control (Ad-GFP) (Figure 5C) and >90% of Ad-GFP exposed fibroblasts expressed GFP. Deletion of *JNK1* increased tropoelastin mRNA three-fold (Figure 5D). To test whether JNK activation suppresses pulmonary fibroblast elastogenesis, we used the JNK activator anisomycin. Anisomycin reduced PND14 wild-type fibroblast tropoelastin mRNA in a dose-dependent manner that was not statistically significant (Figure 5E). Since epithelial or endothelial cell contamination of primary fibroblast cultures could account for the observed differences, we quantitated surfactant protein B and CD31 (Pecam1) mRNA content. Neither marker could be detected in wild type fibroblast culture but the markers were abundant in wild type lung demonstrating that fibroblasts cultures did not have significant epithelial or endothelial cell contamination. Since JNK3 is not expressed in lung [22], these *in vitro* data demonstrate that JNK1 and JNK2 independently suppress pulmonary fibroblast elastogenesis.

### JNK suppresses mRNAs of elastin-associated genes

**Figure 6:**
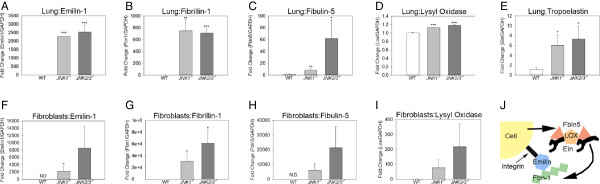
**JNK regulates expression of many elastin-associated genes. (A-C)** The lungs of PND14 JNK-deficient mice contained more emilin-1, fibrillin-1, and fibulin-5 mRNAs than those of wild type mice. **(D)** mRNA of lysyl oxidase was modestly increased in lungs of JNK-deficient mice. **(E)** Tropoelastin mRNA was increased in the lungs of JNK-deficient mice. All lungs were from three PND14 mice from at least two different litters. **(F-I)** Emilin-1, fibrillin-1, fibulin-5, and lysyl oxidase mRNAs were elevated in PND14 JNK-deficient lung fibroblasts compared to wild type. Emilin-1 and fibulin-5 mRNAs were not detected in PND14 wild-type lung fibroblasts, so for statistical comparisons, a Ct value of 40 cycles was assumed. **(J)** A simplified schematic depicts elastin fibril assembly. Tropoelastin (Eln) forms a multimeric complex with binding proteins Fibulin-5 (Fbln5), and the cross-linking enzyme lysyl oxidase (LOX). This multimeric complex then binds to microfibrils composed of fibrillin-1 (Fbn1). Fibrillin-1 assembly is facilitated by emilin which binds to cells via an integrin-binding domain. *p < 0.05, **p < 0.01, ***p < 0.001 by one-way ANOVA. All analyses were performed in triplicate.

Since the mRNAs of many elastin-associated molecules decreased in association with increased lung JNK activity, and since JNK suppressed elastogenesis *in vitro*, we tested whether JNK-deficient lungs had increased mRNA levels of elastin-associated molecules. *JNK1*^*−/−*^ and *JNK2*%*3*^*−/−*^ lungs contained 2,000-fold more emilin-1 (Figure 6A) and 700-fold more fibrillin-1 (Figure 6B) than wild type lungs. *JNK1*^*−/−*^ lungs contained 10-fold and *JNK2*%*3*^*−/−*^ 60-fold more fibulin-5 (Figure 6C), but differences in quantities of lysyl oxidase mRNA were small (Figure 6D). *JNK1*^*−/−*^ and *JNK2*%*3*^*−/−*^ lung contained 6–8 fold more tropoelastin mRNA than wild type (Figure 6E). To confirm that JNK-deficiency itself and not a secondary cell signaling alteration (such as altered mechanical strain or differences in matrix stiffness) was responsible for these differences in elastin-related gene mRNA content, we quantitated these same mRNAs in primary lung fibroblasts. Neither emilin-1 nor fibulin-5 mRNA was detected in wild-type lung fibroblasts but it was present in both *JNK1*^*−/−*^ and *JNK2*%*3*^*−/−*^ lung fibroblasts (Figure 6F%H). Fibrillin-1 mRNA was detected in wild type lung fibroblasts, but mRNA content was several thousand-fold higher in JNK-deficient lung fibroblasts (Figure 6G). Lysyl oxidase mRNA was not statistically different between wild type and JNK-deficient lung fibroblasts (Figure 6I). Tropoelastin mRNA was assessed previously. A schematic depicting how emilin-1, fibrillin-1, fibulin-5, lysyl oxidase, and tropoelastin interact to form elastin fibers is shown in Figure 6J. These data demonstrate that JNK regulates many of the genes involved in elastin microfibril formation and support the concept that JNK suppresses the pulmonary fibroblast elastogenic program.

## Discussion

For the first time, we have demonstrated a role for JNK in suppressing the elastogenic program during the alveolar stage of lung development. JNK activity increased during late alveolar development, and this increased JNK activity was negatively correlated with elastogenic gene mRNAs. JNK-deficiency increased lung elastin content and impaired alveolar septation. Both JNK-deficient lung and lung fibroblasts had increased mRNA levels of elastin-associated genes. Deletion of both *JNK1* and *JNK2* increased tropoelastin mRNA to a greater extent than either individually demonstrating that both isoforms independently regulate elastogenesis. Our observations are consistent with previous reports demonstrating JNK regulation of elastogenesis in the aorta [20] and AP-1 mediated suppression of tropoelastin expression [35]. Phosphorylated c-jun, the readout of the JNK luciferase assay, is a component of the AP-1 transcriptional complex.

We utilized two methods to assess JNK activation during lung development. In addition to comparison of total JNK to phosphorylated JNK, we utilized a pull-down technique developed and validated at the University of California at San Diego in a variety of cell lines with increasing exposures to ultraviolet irradiation—a well described activator of JNK signaling [32]. This technique quantitates JNK phosphorylation of c-Jun. A drawback of this assay is the absence of a loading control which likely accounts for the increased variability in this assay compared to Western blot. Nonetheless, by both Western blot and JNK pull-down, we demonstrated a negative association between lung JNK activity and elastogenic mRNAs.

We have provided strong *in vitro* and *in vivo* loss of function data to support a role for JNK in regulating the elastogenic program in the lung. When assessed in isolation, these effects are clear and strong. However, multiple dynamic processes regulate elastogenesis which likely accounts for larger *in vitro* than *in vivo* effects. JNK activity was reduced in adult lung compared to developing lung. This discrepancy is likely due to reduced transcriptional activation of the elastogenic program in the adult compared to the juvenile. Conceptually, JNK activity may serve as a brake slowing elastogenesis during alveolarization which turns off when the elastogenic program is no longer activated. The localization of pJNK to pulmonary lipofibroblasts is consistent with their reduced elastogenic and fibrogenic potential [31]; although, the fact that lipofibroblasts increase in number at an earlier age than JNK activity levels increase [36] makes a direct relationship between the two unlikely. The mechanism of how JNK suppresses elastogenesis needs to be determined in future studies.

The necessity of elastin band formation for secondary alveolar septation is well-established [4,16]. Both elastin [9] and the lysyl oxidase [37] content are increased in infants with chronic lung disease of prematurity. In mice, elastin haploinsufficiency leads to emphysematous changes [6], and Mammoto *et al.* recently demonstrated that reduced matrix stiffness also impairs alveolarization [38]. Our data and these previously published data demonstrate that alveolar growth requires both a low compliance elastin band and high compliance alveolar walls. Therapies aimed at restoring alveolar growth should seek to normalize this relationship.

In conclusion, we report that JNK suppresses pulmonary fibroblast elastogenesis during the alveolar stage of lung development plays a role in alveolar septation.

## Competing interests

The authors declare that they have no competing interests.

## Authors’ contributions

SL aided in project conceptualization, designed experiments, and performed experiments. HP designed morphometric programs and optimized programs for elastin quantitation. SY performed morphometry and other experiments. BV conceptualized the project, designed experiments, and analyzed data.
